# *tert*-Butyl 4-{[5-(4-chloro­phen­yl)-1-(4-fluoro­phen­yl)-1*H*-pyrazol-3-yl]carbon­yl}piperazine-1-carboxyl­ate

**DOI:** 10.1107/S1600536810038560

**Published:** 2010-09-30

**Authors:** R. Venkat Ragavan, V. Vijayakumar, S. Sarveswari, Seik Weng Ng, Edward R. T. Tiekink

**Affiliations:** a Organic Chemistry Division, School of Advanced Sciences, VIT University, Vellore 632 014, Tamilnadu, India; b Department of Chemistry, University of Malaya, 50603 Kuala Lumpur, Malaysia

## Abstract

In the title pyrazole derivative, C_25_H_26_ClFN_4_O_3_, both benzene rings are twisted out of the plane through the pyrazole ring, with dihedral angles of 67.62 (10) and 27.63 (10)° for the fluoro- and chloro-substituted rings, respectively. The dihedral angle between the two benzene rings is 64.54 (9)°. The piperazine ring (with a chair conformation) is linked to the pyrazole ring *via* a carbonyl spacer and is orientated to lie to one side of the pyrazole plane. In addition to an intra­molecular C—H⋯N contact, there are inter­molecular C—H⋯O inter­actions, which generate a supra­molecular chain with an undulating topology along the *c* axis that is sustained by alternating centrosymmetric ten-membered {⋯HCNCO}_2_ and {⋯HC3O}_2_ synthons.

## Related literature

For the pharmacological potential of pyrazol derivatives, see: Ragavan *et al.* (2009 ▶). For the synthesis, see: Ragavan *et al.* (2010 ▶). For a related structure, see: Samshuddin *et al.* (2010 ▶).
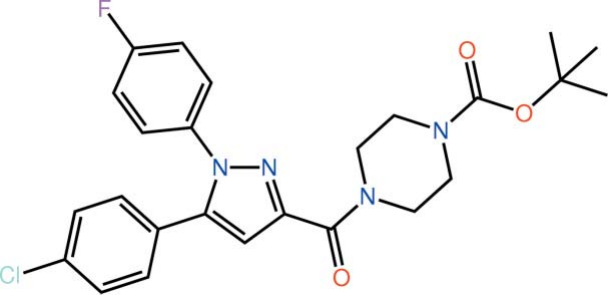


## Experimental

### 

#### Crystal data

C_25_H_26_ClFN_4_O_3_
*M* *_r_* = 484.95
Triclinic, 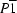

*a* = 6.0568 (5) Å
*b* = 12.0047 (10) Å
*c* = 16.2615 (13) Å
α = 88.852 (1)°
β = 81.206 (1)°
γ = 87.644 (1)°
*V* = 1167.37 (17) Å^3^
*Z* = 2
Mo *K*α radiation
μ = 0.21 mm^−1^
*T* = 100 K
0.35 × 0.10 × 0.10 mm

#### Data collection

Bruker SMART APEX diffractometer
Absorption correction: analytical (FACES; Bruker, 2009 ▶) *T*_min_ = 0.931, *T*_max_ = 0.980
11213 measured reflections
5318 independent reflections
4351 reflections with *I* > 2σ(*I*)
*R*_int_ = 0.030

#### Refinement

*R*[*F* ^2^ > 2σ(*F* ^2^)] = 0.043
*wR*(*F* ^2^) = 0.145
*S* = 1.06
5318 reflections
310 parameters
H-atom parameters constrained
Δρ_max_ = 0.37 e Å^−3^
Δρ_min_ = −0.39 e Å^−3^

### 

Data collection: *APEX2* (Bruker, 2009 ▶); cell refinement: *SAINT* (Bruker, 2009 ▶); data reduction: *SAINT*; program(s) used to solve structure: *SHELXS97* (Sheldrick, 2008 ▶); program(s) used to refine structure: *SHELXL97* (Sheldrick, 2008 ▶); molecular graphics: *ORTEP-3* (Farrugia, 1997 ▶) and *DIAMOND* (Brandenburg, 2006 ▶); software used to prepare material for publication: *publCIF* (Westrip, 2010 ▶).

## Supplementary Material

Crystal structure: contains datablocks global, I. DOI: 10.1107/S1600536810038560/hb5646sup1.cif

Structure factors: contains datablocks I. DOI: 10.1107/S1600536810038560/hb5646Isup2.hkl

Additional supplementary materials:  crystallographic information; 3D view; checkCIF report

## Figures and Tables

**Table 1:** Hydrogen-bond geometry (Å, °)

*D*—H⋯*A*	*D*—H	H⋯*A*	*D*⋯*A*	*D*—H⋯*A*
C17—H17a⋯N2	0.99	2.24	2.950 (2)	128
C14—H14⋯O1^i^	0.95	2.28	3.192 (2)	161
C18—H18a⋯O2^ii^	0.99	2.52	3.223 (2)	128

Symmetry codes: (i) 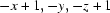; (ii) 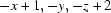.

**Fig. 1.**
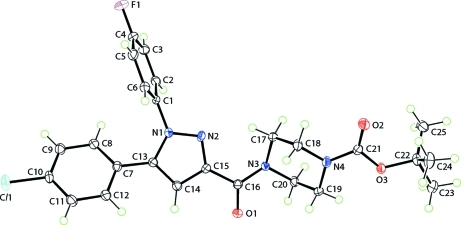
The molecular structure of (I) showing displacement ellipsoids at the 50% probability level.

**Fig. 2.**
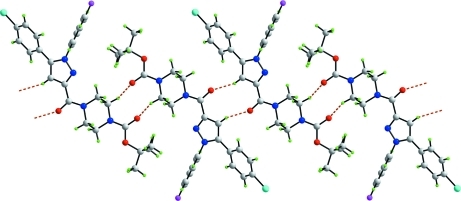
Supramolecular chains aligned along the c axis in (I) mediated by C—H···O interactions (orange dashed lines).

**Fig. 3.**
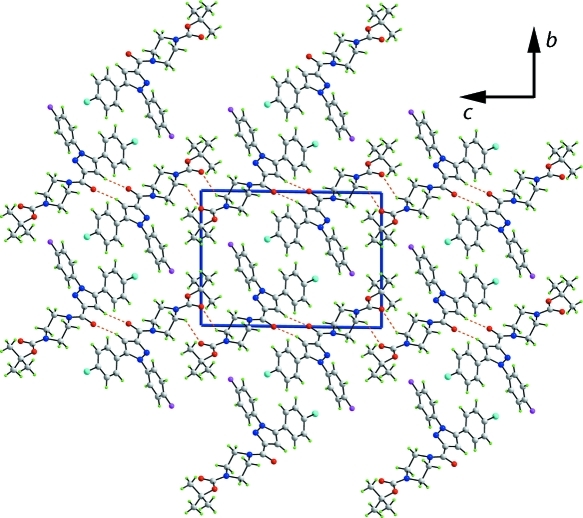
Unit-cell contents shown in projection down the a axis in (I) showing the stacking of layers along the b direction. The C–H···O contacts are shown as orange dashed lines.

## supplementary crystallographic information

### Comment

The anti-bacterial and anti-fungal activities of the azoles are well known and some derivatives are used clinically as anti-microbial agents. However, the emergence of azole-resistant strains of microbes requires the development of new anti-microbial compounds. Pyrazole forms an important class of heterocyclic compounds and many pyrazole derivatives are reported to display a broad spectrum of biological activities, such as anti-inflammatory, anti-fungal, herbicidal, anti-tumour, cytotoxic, and anti-viral activities. Since the high electronegativity of halogens (particularly chlorine and fluorine) in aromatic rings of drug molecules plays an important role in enhancing biological activity, we are interested to have 4-fluoro or 4-chloro substitution in the aryl rings of 1,5-diaryl pyrazoles. As part of our on-going research aimed at the synthesis of new anti-microbial compounds based on pyrazole (Ragavan *et al.*, 2009, 2010) and reflecting our interest in pyrazole structures (Samshuddin *et al.*, 2010), herein we report the crystallographic characterization of a novel pyrazole derivative, (I).

The pyrazole ring in (I), Fig. 1, is planar (r.m.s. deviation = 0.003 Å) and is connected to two halo-substituted benzene rings. Whereas the chloro-substituted ring is slightly twisted out of the plane of the pyrazoyl ring [dihedral angle = 27.63 (10) °], the fluoro-substituted ring is almost orthogonal [dihedral angle = 67.62 (10) °]; the dihedral angle between the two benzene rings = 64.54 (9) °. The ester derivatized piperazine ring (with a chair conformation) is linked to the pyrazoyl ring *via* a carbonyl spacer [the N2—C15—C16—N3 torsion angle = 13.6 (3) °] and is orientated to lie to one side of the pyrazoyl plane. Finally, the ester group is co-planar with the C18—N4—C19 plane as seen in the C18—N4—C21—O2 torsion angle of -0.3 (3) °.

In addition to an intramolecular C—H···N bond, there are two significant intermolecular C—H···O contacts of note, Table 1. The latter lead to the formation of an undulating supramolecular chain along the *c* axis comprising alternating centrosymmetric 10-membered {···HCNCO}_2_ and {···HC_3_O}_2_ synthons, Fig. 2. Chains pack in the *ac* plane and these stack along the *b* axis, Fig. 3.

### Experimental

The compound was synthesized by the literature method (Ragavan *et al.*, 2010). Colourless blocks of (I) were obtained by recrystallization from absolute ethanol; m.pt. 356.1–357.2 K.

### Refinement

Carbon-bound H-atoms were placed in calculated positions (C—H 0.95 to 0.99 Å) and were included in the refinement in the riding model approximation, with *U*_iso_(H) set to 1.2 to 1.5*U*_equiv_(C). In the final refinement two low angle reflections evidently effected by the beam stop were omitted, *i.e*. (010) and (001).

### Crystal data

**Table:**

C_25_H_26_ClFN_4_O_3_	*Z* = 2
*M**_r_* = 484.95	*F*(000) = 508
Triclinic, *P*1	*D*_x_ = 1.380 Mg m^−^^3^
Hall symbol: -P 1	Mo *K*α radiation, λ = 0.71073 Å
*a* = 6.0568 (5) Å	Cell parameters from 3796 reflections
*b* = 12.0047 (10) Å	θ = 3.0–30.6°
*c* = 16.2615 (13) Å	µ = 0.21 mm^−^^1^
α = 88.852 (1)°	*T* = 100 K
β = 81.206 (1)°	Block, colourless
γ = 87.644 (1)°	0.35 × 0.10 × 0.10 mm
*V* = 1167.37 (17) Å^3^	

### Data collection

**Table:**

Bruker SMART APEX diffractometer	5318 independent reflections
Radiation source: fine-focus sealed tube	4351 reflections with *I* > 2σ(*I*)
graphite	*R*_int_ = 0.030
ω scan	θ_max_ = 27.5°, θ_min_ = 2.1°
Absorption correction: analytical (FACES; Bruker, 2009)	*h* = −7→7
*T*_min_ = 0.931, *T*_max_ = 0.980	*k* = −15→15
11213 measured reflections	*l* = −21→21

### Refinement

**Table:**

Refinement on *F*^2^	Primary atom site location: structure-invariant direct methods
Least-squares matrix: full	Secondary atom site location: difference Fourier map
*R*[*F*^2^ > 2σ(*F*^2^)] = 0.043	Hydrogen site location: inferred from neighbouring sites
*wR*(*F*^2^) = 0.145	H-atom parameters constrained
*S* = 1.06	*w* = 1/[σ^2^(*F*_o_^2^) + (0.0829*P*)^2^ + 0.4102*P*] where *P* = (*F*_o_^2^ + 2*F*_c_^2^)/3
5318 reflections	(Δ/σ)_max_ = 0.001
310 parameters	Δρ_max_ = 0.37 e Å^−^^3^
0 restraints	Δρ_min_ = −0.39 e Å^−^^3^

### Special details

**Table:**

Geometry. All s.u.'s (except the s.u. in the dihedral angle between two l.s. planes) are estimated using the full covariance matrix. The cell s.u.'s are taken into account individually in the estimation of s.u.'s in distances, angles and torsion angles; correlations between s.u.'s in cell parameters are only used when they are defined by crystal symmetry. An approximate (isotropic) treatment of cell s.u.'s is used for estimating s.u.'s involving l.s. planes.
Refinement. Refinement of *F*^2^ against ALL reflections. The weighted *R*-factor *wR* and goodness of fit *S* are based on *F*^2^, conventional *R*-factors *R* are based on *F*, with *F* set to zero for negative *F*^2^. The threshold expression of *F*^2^ > 2σ(*F*^2^) is used only for calculating *R*-factors(gt) *etc*. and is not relevant to the choice of reflections for refinement. *R*-factors based on *F*^2^ are statistically about twice as large as those based on *F*, and *R*- factors based on ALL data will be even larger.

### Fractional atomic coordinates and isotropic or equivalent isotropic displacement parameters (Å^2^)

**Table:**

	*x*	*y*	*z*	*U*_iso_*/*U*_eq_	
Cl1	−0.54699 (8)	0.35562 (4)	0.36749 (3)	0.02774 (15)	
F1	−0.3361 (2)	0.60200 (11)	0.83637 (8)	0.0351 (3)	
O1	0.6767 (2)	−0.00368 (11)	0.59753 (8)	0.0216 (3)	
O2	0.6127 (2)	−0.13676 (12)	1.00046 (8)	0.0223 (3)	
O3	0.9285 (2)	−0.21367 (11)	0.92542 (8)	0.0186 (3)	
N1	0.1504 (2)	0.26137 (12)	0.66173 (9)	0.0155 (3)	
N2	0.3142 (2)	0.20929 (13)	0.69823 (10)	0.0167 (3)	
N3	0.6639 (2)	0.04516 (13)	0.73200 (9)	0.0150 (3)	
N4	0.6836 (3)	−0.11496 (14)	0.86022 (10)	0.0202 (3)	
C1	0.0220 (3)	0.35009 (14)	0.70660 (10)	0.0148 (3)	
C2	0.1251 (3)	0.44859 (15)	0.71731 (11)	0.0174 (4)	
H2	0.2778	0.4572	0.6948	0.021*	
C3	0.0030 (3)	0.53487 (16)	0.76141 (12)	0.0225 (4)	
H3	0.0699	0.6033	0.7692	0.027*	
C4	−0.2166 (3)	0.51834 (17)	0.79337 (12)	0.0226 (4)	
C5	−0.3210 (3)	0.42072 (17)	0.78344 (12)	0.0213 (4)	
H5	−0.4732	0.4122	0.8067	0.026*	
C6	−0.2004 (3)	0.33534 (16)	0.73909 (11)	0.0183 (4)	
H6	−0.2690	0.2675	0.7310	0.022*	
C7	−0.0359 (3)	0.25317 (15)	0.53333 (11)	0.0154 (4)	
C8	−0.1139 (3)	0.36381 (15)	0.52973 (11)	0.0180 (4)	
H8	−0.0618	0.4178	0.5634	0.022*	
C9	−0.2670 (3)	0.39586 (16)	0.47737 (12)	0.0199 (4)	
H9	−0.3181	0.4715	0.4747	0.024*	
C10	−0.3444 (3)	0.31675 (16)	0.42920 (11)	0.0183 (4)	
C11	−0.2662 (3)	0.20696 (16)	0.43007 (12)	0.0212 (4)	
H11	−0.3183	0.1535	0.3960	0.025*	
C12	−0.1106 (3)	0.17607 (16)	0.48145 (11)	0.0188 (4)	
H12	−0.0536	0.1011	0.4814	0.023*	
C13	0.1254 (3)	0.21355 (14)	0.58746 (11)	0.0147 (3)	
C14	0.2801 (3)	0.12616 (15)	0.57692 (11)	0.0166 (4)	
H14	0.3060	0.0757	0.5319	0.020*	
C15	0.3920 (3)	0.12677 (14)	0.64627 (11)	0.0150 (3)	
C16	0.5851 (3)	0.04965 (14)	0.65783 (11)	0.0149 (3)	
C17	0.5275 (3)	0.06276 (15)	0.81360 (11)	0.0168 (4)	
H17A	0.3856	0.1031	0.8066	0.020*	
H17B	0.6083	0.1087	0.8482	0.020*	
C18	0.4778 (3)	−0.04902 (16)	0.85671 (12)	0.0194 (4)	
H18A	0.3988	−0.0365	0.9139	0.023*	
H18B	0.3788	−0.0904	0.8261	0.023*	
C19	0.8221 (3)	−0.13264 (16)	0.77930 (11)	0.0190 (4)	
H19A	0.7429	−0.1786	0.7441	0.023*	
H19B	0.9640	−0.1726	0.7869	0.023*	
C20	0.8709 (3)	−0.02034 (16)	0.73720 (11)	0.0180 (4)	
H20A	0.9664	0.0214	0.7690	0.022*	
H20B	0.9537	−0.0320	0.6805	0.022*	
C21	0.7328 (3)	−0.15410 (15)	0.93439 (11)	0.0165 (4)	
C22	1.0055 (3)	−0.26720 (16)	0.99898 (11)	0.0191 (4)	
C23	1.2187 (3)	−0.32815 (17)	0.96125 (13)	0.0250 (4)	
H23A	1.1842	−0.3848	0.9231	0.038*	
H23B	1.2900	−0.3642	1.0056	0.038*	
H23C	1.3205	−0.2750	0.9306	0.038*	
C24	0.8349 (3)	−0.34893 (18)	1.03985 (14)	0.0285 (5)	
H24A	0.7967	−0.3989	0.9977	0.043*	
H24B	0.6998	−0.3076	1.0655	0.043*	
H24C	0.8984	−0.3928	1.0826	0.043*	
C25	1.0532 (3)	−0.17857 (18)	1.05902 (12)	0.0236 (4)	
H25A	1.1630	−0.1278	1.0301	0.035*	
H25B	1.1128	−0.2146	1.1061	0.035*	
H25C	0.9145	−0.1363	1.0795	0.035*	

### Atomic displacement parameters (Å^2^)

**Table:**

	*U*^11^	*U*^22^	*U*^33^	*U*^12^	*U*^13^	*U*^23^
Cl1	0.0254 (3)	0.0297 (3)	0.0319 (3)	−0.0024 (2)	−0.0172 (2)	0.0069 (2)
F1	0.0380 (7)	0.0319 (7)	0.0337 (7)	0.0169 (6)	−0.0032 (6)	−0.0137 (6)
O1	0.0250 (7)	0.0246 (7)	0.0150 (6)	0.0078 (6)	−0.0038 (5)	−0.0048 (5)
O2	0.0206 (7)	0.0289 (7)	0.0157 (7)	0.0027 (5)	0.0011 (5)	0.0025 (6)
O3	0.0179 (6)	0.0224 (7)	0.0154 (6)	0.0036 (5)	−0.0036 (5)	0.0020 (5)
N1	0.0174 (7)	0.0153 (7)	0.0146 (7)	0.0012 (6)	−0.0054 (6)	0.0004 (6)
N2	0.0159 (7)	0.0155 (7)	0.0192 (8)	0.0029 (6)	−0.0057 (6)	0.0019 (6)
N3	0.0150 (7)	0.0178 (7)	0.0117 (7)	0.0035 (6)	−0.0017 (5)	0.0005 (6)
N4	0.0211 (8)	0.0230 (8)	0.0148 (7)	0.0073 (6)	−0.0001 (6)	0.0034 (6)
C1	0.0170 (8)	0.0150 (8)	0.0123 (8)	0.0057 (6)	−0.0037 (6)	−0.0006 (6)
C2	0.0180 (8)	0.0198 (9)	0.0147 (8)	−0.0006 (7)	−0.0031 (7)	0.0003 (7)
C3	0.0299 (10)	0.0169 (9)	0.0221 (9)	0.0012 (8)	−0.0090 (8)	−0.0037 (7)
C4	0.0274 (10)	0.0216 (10)	0.0185 (9)	0.0107 (8)	−0.0049 (8)	−0.0044 (7)
C5	0.0173 (9)	0.0276 (10)	0.0182 (9)	0.0042 (7)	−0.0018 (7)	0.0011 (8)
C6	0.0197 (9)	0.0179 (9)	0.0178 (9)	0.0005 (7)	−0.0043 (7)	0.0002 (7)
C7	0.0141 (8)	0.0172 (9)	0.0145 (8)	0.0000 (6)	−0.0015 (6)	0.0024 (7)
C8	0.0202 (9)	0.0176 (9)	0.0166 (8)	−0.0001 (7)	−0.0040 (7)	−0.0003 (7)
C9	0.0211 (9)	0.0193 (9)	0.0194 (9)	0.0007 (7)	−0.0043 (7)	0.0024 (7)
C10	0.0150 (8)	0.0245 (10)	0.0157 (8)	0.0009 (7)	−0.0048 (7)	0.0044 (7)
C11	0.0236 (9)	0.0215 (10)	0.0203 (9)	−0.0035 (7)	−0.0083 (7)	−0.0007 (7)
C12	0.0226 (9)	0.0185 (9)	0.0153 (8)	0.0005 (7)	−0.0037 (7)	0.0007 (7)
C13	0.0161 (8)	0.0147 (8)	0.0134 (8)	−0.0002 (6)	−0.0023 (6)	−0.0005 (6)
C14	0.0185 (8)	0.0160 (9)	0.0155 (8)	−0.0010 (7)	−0.0033 (7)	0.0002 (7)
C15	0.0160 (8)	0.0127 (8)	0.0162 (8)	0.0017 (6)	−0.0028 (7)	0.0000 (7)
C16	0.0159 (8)	0.0139 (8)	0.0148 (8)	−0.0005 (6)	−0.0019 (6)	0.0005 (7)
C17	0.0184 (8)	0.0183 (9)	0.0126 (8)	0.0042 (7)	−0.0004 (7)	−0.0016 (7)
C18	0.0167 (8)	0.0233 (10)	0.0169 (9)	0.0040 (7)	−0.0008 (7)	0.0039 (7)
C19	0.0210 (9)	0.0204 (9)	0.0141 (8)	0.0087 (7)	−0.0006 (7)	−0.0021 (7)
C20	0.0142 (8)	0.0243 (10)	0.0152 (8)	0.0043 (7)	−0.0024 (7)	0.0013 (7)
C21	0.0160 (8)	0.0139 (8)	0.0199 (9)	0.0003 (6)	−0.0039 (7)	0.0001 (7)
C22	0.0194 (9)	0.0220 (9)	0.0167 (9)	0.0008 (7)	−0.0061 (7)	0.0041 (7)
C23	0.0226 (10)	0.0252 (10)	0.0276 (10)	0.0059 (8)	−0.0070 (8)	0.0011 (8)
C24	0.0262 (10)	0.0279 (11)	0.0319 (11)	−0.0026 (8)	−0.0070 (9)	0.0127 (9)
C25	0.0196 (9)	0.0335 (11)	0.0175 (9)	0.0018 (8)	−0.0030 (7)	−0.0020 (8)

### Geometric parameters (Å, °)

**Table:**

Cl1—C10	1.7439 (18)	C9—H9	0.9500
F1—C4	1.355 (2)	C10—C11	1.383 (3)
O1—C16	1.228 (2)	C11—C12	1.387 (2)
O2—C21	1.218 (2)	C11—H11	0.9500
O3—C21	1.349 (2)	C12—H12	0.9500
O3—C22	1.475 (2)	C13—C14	1.373 (2)
N1—N2	1.357 (2)	C14—C15	1.402 (2)
N1—C13	1.379 (2)	C14—H14	0.9500
N1—C1	1.436 (2)	C15—C16	1.494 (2)
N2—C15	1.338 (2)	C17—C18	1.521 (3)
N3—C16	1.363 (2)	C17—H17A	0.9900
N3—C20	1.465 (2)	C17—H17B	0.9900
N3—C17	1.466 (2)	C18—H18A	0.9900
N4—C21	1.356 (2)	C18—H18B	0.9900
N4—C18	1.457 (2)	C19—C20	1.519 (3)
N4—C19	1.463 (2)	C19—H19A	0.9900
C1—C6	1.387 (3)	C19—H19B	0.9900
C1—C2	1.386 (2)	C20—H20A	0.9900
C2—C3	1.392 (3)	C20—H20B	0.9900
C2—H2	0.9500	C22—C23	1.509 (3)
C3—C4	1.373 (3)	C22—C24	1.523 (3)
C3—H3	0.9500	C22—C25	1.524 (3)
C4—C5	1.378 (3)	C23—H23A	0.9800
C5—C6	1.382 (3)	C23—H23B	0.9800
C5—H5	0.9500	C23—H23C	0.9800
C6—H6	0.9500	C24—H24A	0.9800
C7—C8	1.394 (3)	C24—H24B	0.9800
C7—C12	1.398 (2)	C24—H24C	0.9800
C7—C13	1.471 (2)	C25—H25A	0.9800
C8—C9	1.390 (2)	C25—H25B	0.9800
C8—H8	0.9500	C25—H25C	0.9800
C9—C10	1.384 (3)		
			
C21—O3—C22	119.64 (14)	C14—C15—C16	124.05 (16)
N2—N1—C13	112.50 (14)	O1—C16—N3	121.74 (16)
N2—N1—C1	117.44 (14)	O1—C16—C15	118.02 (15)
C13—N1—C1	129.91 (14)	N3—C16—C15	120.11 (15)
C15—N2—N1	104.23 (14)	N3—C17—C18	109.81 (14)
C16—N3—C20	118.19 (14)	N3—C17—H17A	109.7
C16—N3—C17	125.08 (14)	C18—C17—H17A	109.7
C20—N3—C17	112.72 (14)	N3—C17—H17B	109.7
C21—N4—C18	120.10 (15)	C18—C17—H17B	109.7
C21—N4—C19	125.57 (15)	H17A—C17—H17B	108.2
C18—N4—C19	114.33 (14)	N4—C18—C17	110.80 (15)
C6—C1—C2	121.44 (16)	N4—C18—H18A	109.5
C6—C1—N1	119.72 (16)	C17—C18—H18A	109.5
C2—C1—N1	118.84 (16)	N4—C18—H18B	109.5
C1—C2—C3	119.41 (17)	C17—C18—H18B	109.5
C1—C2—H2	120.3	H18A—C18—H18B	108.1
C3—C2—H2	120.3	N4—C19—C20	109.09 (15)
C4—C3—C2	118.21 (18)	N4—C19—H19A	109.9
C4—C3—H3	120.9	C20—C19—H19A	109.9
C2—C3—H3	120.9	N4—C19—H19B	109.9
F1—C4—C3	118.44 (18)	C20—C19—H19B	109.9
F1—C4—C5	118.57 (18)	H19A—C19—H19B	108.3
C3—C4—C5	122.99 (18)	N3—C20—C19	111.15 (14)
C4—C5—C6	118.84 (18)	N3—C20—H20A	109.4
C4—C5—H5	120.6	C19—C20—H20A	109.4
C6—C5—H5	120.6	N3—C20—H20B	109.4
C5—C6—C1	119.12 (17)	C19—C20—H20B	109.4
C5—C6—H6	120.4	H20A—C20—H20B	108.0
C1—C6—H6	120.4	O2—C21—O3	124.94 (16)
C8—C7—C12	118.48 (16)	O2—C21—N4	123.32 (17)
C8—C7—C13	123.44 (16)	O3—C21—N4	111.74 (15)
C12—C7—C13	118.06 (16)	O3—C22—C23	102.28 (14)
C9—C8—C7	120.63 (17)	O3—C22—C24	110.27 (15)
C9—C8—H8	119.7	C23—C22—C24	110.76 (17)
C7—C8—H8	119.7	O3—C22—C25	109.96 (15)
C10—C9—C8	119.52 (17)	C23—C22—C25	110.84 (16)
C10—C9—H9	120.2	C24—C22—C25	112.29 (17)
C8—C9—H9	120.2	C22—C23—H23A	109.5
C11—C10—C9	121.10 (16)	C22—C23—H23B	109.5
C11—C10—Cl1	119.40 (14)	H23A—C23—H23B	109.5
C9—C10—Cl1	119.49 (14)	C22—C23—H23C	109.5
C10—C11—C12	118.99 (17)	H23A—C23—H23C	109.5
C10—C11—H11	120.5	H23B—C23—H23C	109.5
C12—C11—H11	120.5	C22—C24—H24A	109.5
C11—C12—C7	121.22 (17)	C22—C24—H24B	109.5
C11—C12—H12	119.4	H24A—C24—H24B	109.5
C7—C12—H12	119.4	C22—C24—H24C	109.5
C14—C13—N1	105.62 (15)	H24A—C24—H24C	109.5
C14—C13—C7	129.58 (16)	H24B—C24—H24C	109.5
N1—C13—C7	124.79 (16)	C22—C25—H25A	109.5
C13—C14—C15	105.83 (16)	C22—C25—H25B	109.5
C13—C14—H14	127.1	H25A—C25—H25B	109.5
C15—C14—H14	127.1	C22—C25—H25C	109.5
N2—C15—C14	111.80 (15)	H25A—C25—H25C	109.5
N2—C15—C16	124.02 (15)	H25B—C25—H25C	109.5
			
C13—N1—N2—C15	−0.52 (19)	N1—C13—C14—C15	−0.33 (19)
C1—N1—N2—C15	175.46 (15)	C7—C13—C14—C15	179.05 (17)
N2—N1—C1—C6	−110.40 (18)	N1—N2—C15—C14	0.29 (19)
C13—N1—C1—C6	64.8 (2)	N1—N2—C15—C16	176.34 (16)
N2—N1—C1—C2	68.8 (2)	C13—C14—C15—N2	0.0 (2)
C13—N1—C1—C2	−116.1 (2)	C13—C14—C15—C16	−176.02 (16)
C6—C1—C2—C3	−0.2 (3)	C20—N3—C16—O1	3.6 (3)
N1—C1—C2—C3	−179.36 (16)	C17—N3—C16—O1	−152.18 (17)
C1—C2—C3—C4	0.5 (3)	C20—N3—C16—C15	−172.10 (15)
C2—C3—C4—F1	179.97 (16)	C17—N3—C16—C15	32.1 (2)
C2—C3—C4—C5	−0.3 (3)	N2—C15—C16—O1	−162.19 (17)
F1—C4—C5—C6	179.53 (16)	C14—C15—C16—O1	13.4 (3)
C3—C4—C5—C6	−0.3 (3)	N2—C15—C16—N3	13.6 (3)
C4—C5—C6—C1	0.5 (3)	C14—C15—C16—N3	−170.78 (16)
C2—C1—C6—C5	−0.3 (3)	C16—N3—C17—C18	101.11 (19)
N1—C1—C6—C5	178.85 (15)	C20—N3—C17—C18	−55.79 (19)
C12—C7—C8—C9	1.7 (3)	C21—N4—C18—C17	124.53 (18)
C13—C7—C8—C9	−179.81 (17)	C19—N4—C18—C17	−55.4 (2)
C7—C8—C9—C10	0.8 (3)	N3—C17—C18—N4	53.54 (19)
C8—C9—C10—C11	−2.3 (3)	C21—N4—C19—C20	−124.91 (19)
C8—C9—C10—Cl1	176.78 (14)	C18—N4—C19—C20	55.0 (2)
C9—C10—C11—C12	1.2 (3)	C16—N3—C20—C19	−101.57 (18)
Cl1—C10—C11—C12	−177.88 (14)	C17—N3—C20—C19	57.07 (19)
C10—C11—C12—C7	1.4 (3)	N4—C19—C20—N3	−54.33 (19)
C8—C7—C12—C11	−2.9 (3)	C22—O3—C21—O2	2.6 (3)
C13—C7—C12—C11	178.60 (17)	C22—O3—C21—N4	−177.38 (15)
N2—N1—C13—C14	0.5 (2)	C18—N4—C21—O2	−0.3 (3)
C1—N1—C13—C14	−174.80 (17)	C19—N4—C21—O2	179.57 (17)
N2—N1—C13—C7	−178.88 (16)	C18—N4—C21—O3	179.63 (15)
C1—N1—C13—C7	5.8 (3)	C19—N4—C21—O3	−0.5 (3)
C8—C7—C13—C14	−151.42 (19)	C21—O3—C22—C23	177.25 (15)
C12—C7—C13—C14	27.0 (3)	C21—O3—C22—C24	59.4 (2)
C8—C7—C13—N1	27.9 (3)	C21—O3—C22—C25	−64.9 (2)
C12—C7—C13—N1	−153.69 (17)		

### Hydrogen-bond geometry (Å, °)

**Table:**

*D*—H···*A*	*D*—H	H···*A*	*D*···*A*	*D*—H···*A*
C17—H17a···N2	0.99	2.24	2.950 (2)	128
C14—H14···O1^i^	0.95	2.28	3.192 (2)	161
C18—H18a···O2^ii^	0.99	2.52	3.223 (2)	128

Symmetry codes: (i) −*x*+1, −*y*, −*z*+1; (ii) −*x*+1, −*y*, −*z*+2.

## References

[bb1] Brandenburg, K. (2006). *DIAMOND* Crystal Impact GbR, Bonn, Germany.

[bb2] Bruker (2009). *FACES*, *APEX2* and *SAINT* Bruker AXS Inc., Madison, Wisconsin, USA.

[bb3] Farrugia, L. J. (1997). *J. Appl. Cryst.* **30**, 565.

[bb4] Ragavan, R. V., Vijayakumar, V. & Kumari, N. S. (2009). *Eur. J. Med. Chem.* **44**, 3852–3857.

[bb5] Ragavan, R. V., Vijayakumar, V. & Kumari, N. S. (2010). *Eur. J. Med. Chem.* **45**, 1173–1180. 10.1016/j.ejmech.2009.12.042 20053480

[bb6] Samshuddin, S., Narayana, B., Yathirajan, H. S., Safwan, A. P. & Tiekink, E. R. T. (2010). *Acta Cryst.* E**66**, o1279–o1280. 10.1107/S1600536810015795 PMC2979444 21579379

[bb7] Sheldrick, G. M. (2008). *Acta Cryst.* A**64**, 112–122. 10.1107/S0108767307043930 18156677

[bb8] Westrip, S. P. (2010). *J. Appl. Cryst.* **43**, 920–925.

